# A Rare Case of Anterior Wall Combined With Inferior Wall Wellens Syndrome

**DOI:** 10.1111/anec.70052

**Published:** 2025-01-27

**Authors:** Lei Zhang

**Affiliations:** ^1^ Department of Electrocardiogram, The First Affiliated Hospital Zhejiang University School of Medicine Hangzhou China

**Keywords:** electrocardiogram, inferior wall, non ST‐segment elevation myocardial infarction, Wellens syndrome

## Abstract

This article describes a woman who presented to the hospital with recurrent chest pain. The electrocardiogram revealed positive and negative biphasic T waves in the anterior and inferior leads, which subsequently deepened. Upon recurrence of chest pain, the T waves reverted to upright. Coronary angiography indicated the presence of three‐vessel coronary artery disease. The occurrence of Wellens' T wave sign in the precordial leads frequently suggests left anterior descending artery disease, while the presence of positive and negative biphasic T waves in the inferior wall leads may also indicate lesions in the right coronary artery or left circumflex artery.

## Graphical abstract

The evolution process of Wellens syndrome with anterior and inferior wall.

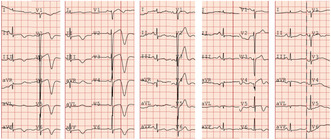

## 1 Case Presentation

**FIGURE 1:**
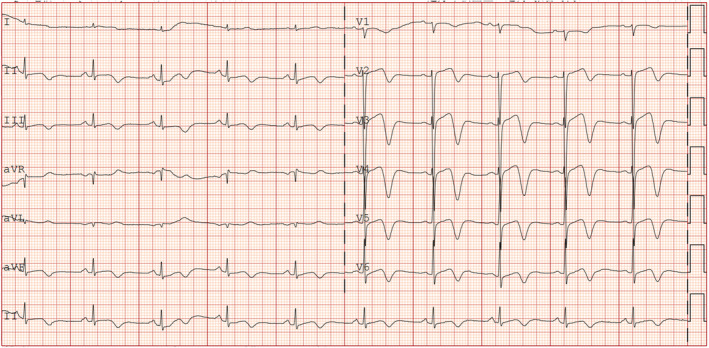
90 min after the onset of chest pain, obvious positive and negative biphasic T waves appeared in leads V2–V6, and at the same time, positive and negative biphasic T waves also appeared in the inferior leads.

**FIGURE 2:**
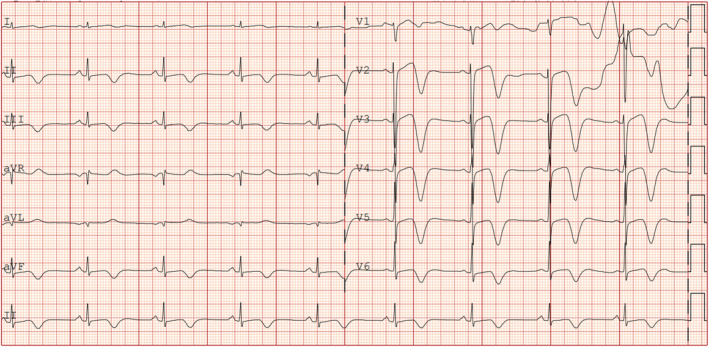
After 60 min, more obvious positive and negative biphasic T waves can be seen in the V2–V6 leads and the inferior leads.

**FIGURE 3:**
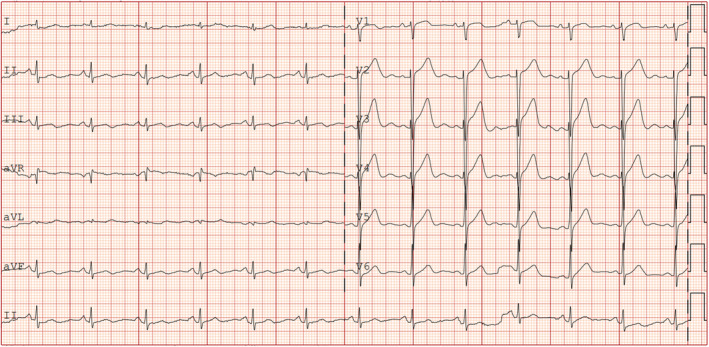
Chest pain recurred 6 h later, and the T waves in leads V2–V6 and inferior leads changed from positive and negative biphasic to upright.

**FIGURE 4:**
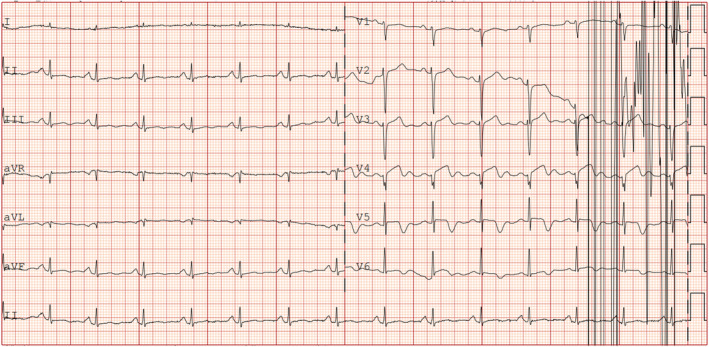
The left side of the chest pain recurred during hospitalization on the second day. It could be seen that the R wave decreased in leads V2–V4, and the ST segment in leads V3–V6 showed a dorsal elevation of 0.05–0.2 mV, accompanied by convulsions in leads V5 and V6 T wave inversion.

A 69‐year‐old woman with a history of hypertension and diabetes presented with recurring left chest pain that began 1 week prior, occurring without obvious triggers. Each episode lasted approximately 5–15 min and was not relieved by rest. Four hours after the initial onset, she experienced another episode of chest pain lasting about 10 min, accompanied by dyspnea and profuse sweating. Upon arrival at the emergency room for evaluation, her blood pressure was measured at 161/109 mmHg, and her pulse was 80 beats per minute. At this point, approximately 90 min had elapsed since the last episode of chest pain, which had since resolved. The initial electrocardiogram (Figure 1) revealed visible V2–V6 leads, exhibiting pronounced positive and negative biphasic T waves, which were also present in the inferior leads. Troponin levels were recorded at 0.019 ng/mL. A follow‐up electrocardiogram was conducted 60 min later (Figure 2), showing more pronounced positive and negative biphasic T waves in the V2‐V6 leads and inferior wall leads, with troponin levels rising to 0.03 ng/mL. Following admission to the hospital, elective coronary angiography was planned. Chest pain recurred 6 h later, prompting a review of the electrocardiogram (Figure 3). It was observed that the T waves in leads V2–V6 and the inferior leads transitioned from positive and negative biphasic to upright. However, the left‐sided chest pain recurred the following day during hospitalization, prompting an immediate electrocardiogram (Figure 4). This showed a decrease in the R wave amplitude in leads V2–V4, with the ST segment in leads V3–V6 exhibiting dorsal elevation of 0.05–0.2 mV. Additionally, T wave inversion was noted in leads V5 and V6.

**FIGURE 5:**
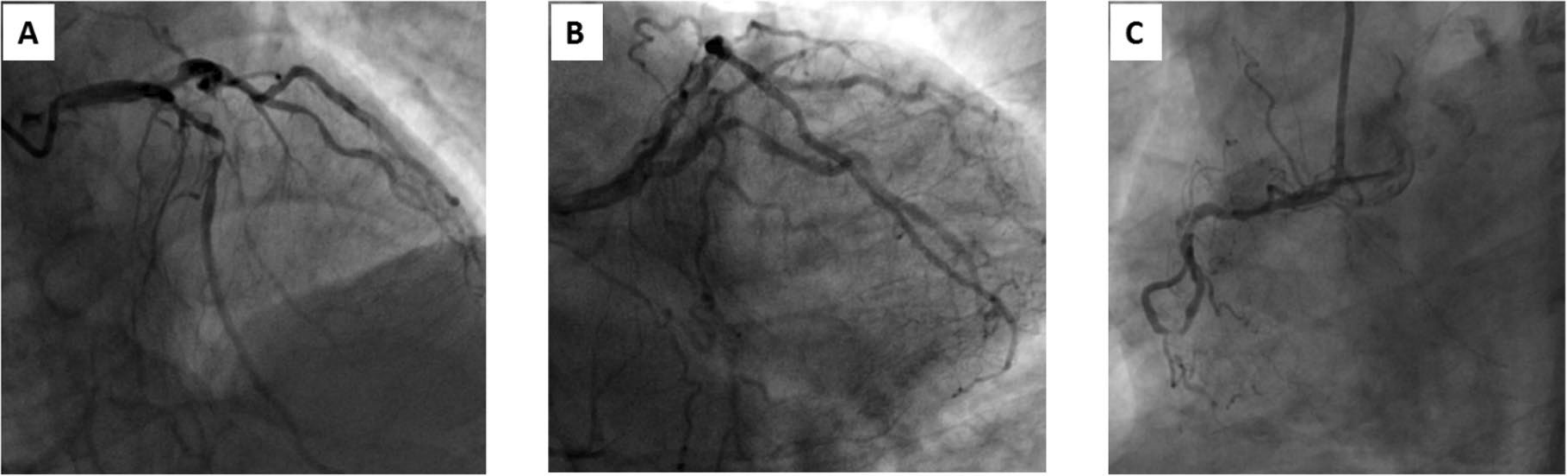
(A) The proximal stenosis of the LAD artery is 70%, and the middle stenosis is 90%. (B) The proximal stenosis of the LCX artery is 80%. (C) The proximal stenosis of the RCA is 60%, and the middle stenosis is 80%.

**FIGURE 6:**
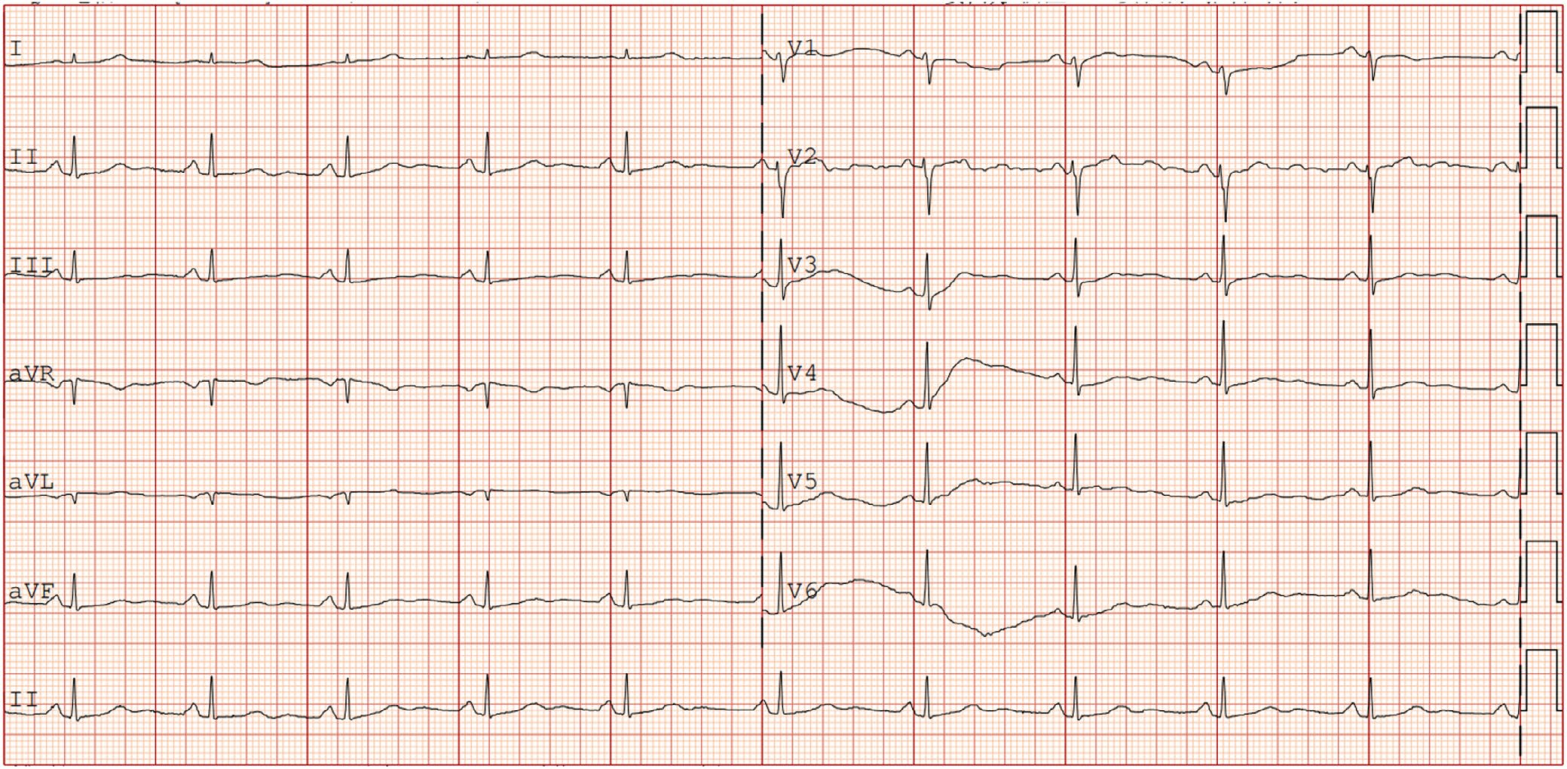
Five hours after post‐angioplasty, the ST segment in leads V3–V6 returned to baseline, and the T waves in leads V5 and V6 returned to upright position.

Coronary angiography (Figure 5) revealed severe lesions in three branches of the coronary arteries: the proximal LAD exhibited 70% stenosis, while the mid‐segment showed 90% stenosis. The proximal LCX had 80% stenosis, and the RCA presented with proximal 60% stenosis and mid‐segment stenosis of 80%. The patient subsequently underwent coronary angioplasty and the implantation of a drug‐eluting stent. Five hours post‐angioplasty, an electrocardiogram (Figure 6) was performed, revealing that the ST segment in leads V3–V6 had returned to baseline, and the T waves in leads V5 and V6 return to upright. He was discharged on the 5th postoperative day without any complications.

## 2 Discussion

Wellens syndrome was first reported by Zwaan, Bar, and Wellens (1982) in 1982. Among 145 patients with unstable angina, they identified a distinctive T wave change in 26 cases (17.9%), of which 12 cases (8.3%) required hospitalization. Extensive anterior wall myocardial infarction occurred during this period. Tandy, Bottomy, and Lewis (1999) classified Wellens syndrome into two types: bidirectional T wave and deeply inverted T wave. In 1983, Haines et al. (1983) reported on 118 patients with unstable angina, 29 of whom exhibited new T wave inversion (≥ 2 leads) in the anterior wall leads; of these, 26 (86%) had LAD stenosis greater than 70%, confirmed by coronary angiography. The sensitivity of T wave changes in chest leads for predicting LAD lesions is 69%, while the specificity is 89%, and the positive predictive value is 86%, further confirming that such T wave changes are associated with severe stenosis in the proximal and middle segments of the LAD. Since then, an increasing number of cases and literature have reported this abnormal T wave pattern on the electrocardiogram, leading to its alternative designation as “anterior descending T wave syndrome.” Subsequently, the clinical characteristics of this specific electrocardiogram pattern were summarized as follows (de Zwaan et al. 1989): (1) a history of chest pain attacks, with no characteristic T wave changes during episodes of chest pain. (2) After the resolution of chest pain for several hours or days, biphasic T waves or symmetrical deeply inverted T waves appear in the precordial leads. (3) Absence of pathological Q waves and ST segment elevation in the precordial leads. (4) Myocardial injury markers are normal or only slightly elevated. (5) This strongly suggests that the culprit vessel is proximal LAD artery stenosis.

For many years, clinicians believed that the Wellens T wave sign was confined to the precordial leads and primarily associated with lesions in the LAD. However, as additional cases were reported, the presence of the Wellens T wave sign in inferior and posterior leads (Lindow, Pahlm, and Nikus 2016; Driver, Shroff, and Smith 2017; Kim et al. 2020; Piña‐Paz and Singh 2021) was also documented, indicating that lesions can arise from the RCA or the LCX. However, during the evolution of the ECG in these cases, the changes were confined to either the anterior wall, inferior wall, or posterior wall, representing unilateral ventricular wall involvement. To date, the Wellens T wave sign resulting from a combination of the anterior wall with either the inferior wall or posterior wall has not been reported. In addition to the evolution of the Wellens T wave sign in the anterior chest leads of this patient, the inferior leads also exhibited a pattern consistent with the typical evolution of the Wellens T wave sign, along with a characteristic ECG manifestation of the transition to ST‐segment elevation myocardial infarction (STEMI). Furthermore, coronary angiography revealed significant stenosis not only in the LAD but also in the RCA and the LCX. This is the reason the Wellens T wave sign is observed on both the anterior and inferior walls.

The specific mechanism underlying Wellens syndrome remains incompletely understood. Currently, most scholars posit that myocardial stunning (Stankovic et al. 2017), resulting from ischemia–reperfusion injury, is the primary cause of the observed T wave changes and their evolution. Myocardial stunning represents a state in which the myocardium is on the brink of necrosis, with the potential to progress to coronary artery occlusion and myocardial infarction. However, it can also revert to normal function following the improvement of ischemia. During an angina pectoris episode, a complete or nearly complete occlusion of the coronary arteries leads to significant myocardial ischemia. Subsequently, the coronary arteries may rapidly recanalize, or adequate blood supply may be restored through collateral circulation, resulting in myocardial reperfusion accompanied by reperfusion injury. At this stage, there is often no corresponding ST‐segment elevation following coronary artery recanalization; instead, significant repolarization abnormalities manifest, characterized by bidirectional or inverted T waves. As myocardial function gradually normalizes over days or weeks, these repolarization abnormalities also progressively resolve, leading to a gradual return of the T wave to an upright position. Some scholars also believe that myocardial tissue edema is a significant contributor to the Wellens T wave sign. Migliore et al. (2011) reported that four patients exhibiting the Wellens T wave sign underwent cardiac magnetic resonance imaging, which revealed myocardial edema in the middle and lower segments of the anterior ventricular septum, along with segmental left ventricular functional abnormalities. After a follow‐up period of 6–8 weeks, the T wave of the electrocardiogram returned to normal, and magnetic resonance imaging confirmed the resolution of myocardial edema in the middle and lower segments of the anterior ventricular septum.

A defining characteristic of patients exhibiting Wellens' T wave sign is the dissociation between the severity of chest pain and the electrocardiogram findings. Notably, when patients experience intense chest pain, the T wave may be upright, and the electrocardiogram can appear nearly normal. However, once the chest pain subsides, positive and negative biphasic or inverted T waves may emerge in the corresponding leads. This phenomenon can pose a challenge in the assessment of patients presenting with chest pain in both outpatient and emergency settings, highlighting the critical importance of reviewing the electrocardiogram after the resolution of chest pain.

## 3 Conclusion

In summary, the significance of T wave changes in Wellens syndrome lies in their ability to identify patients at high risk for unstable angina or non‐ST‐segment elevation myocardial infarction, pinpoint the affected coronary arteries, and indicate the need for early coronary intervention. These patients are at a heightened risk of developing acute ST‐segment elevation myocardial infarction and should undergo coronary intervention or surgical bypass as soon as possible to prevent further deterioration of their condition. Patients suspected of Wellens syndrome should not undergo exercise tests or other cardiac stress tests, as these may exacerbate the condition and lead to acute myocardial infarction or even sudden death. Particularly in cases where Wellens T wave signs are present in multiple ECG leads simultaneously, this finding is highly indicative of double or triple coronary artery disease and warrants significant attention.

## Author Contributions

Lei Zhang performed ECG measurements, the analysis with discussion and the initial manuscript preparation.

## Conflicts of Interest

The author declares no conflicts of interest.

## Data Availability

The data that support the findings of this study are openly available in [repository name] at [DOI], reference number [reference number].
